# Mutation accumulation opposes polymorphism: supergenes and the curious case of balanced lethals

**DOI:** 10.1098/rstb.2021.0199

**Published:** 2022-08-01

**Authors:** Emma L. Berdan, Alexandre Blanckaert, Roger K. Butlin, Thomas Flatt, Tanja Slotte, Ben Wielstra

**Affiliations:** ^1^ Naturalis Biodiversity Center, PO Box 9517, 2300 RA, Leiden, The Netherlands; ^2^ Institute of Biology Leiden, Leiden University, PO Box 9505, 2300 RA, Leiden, The Netherlands; ^3^ Tjarnö Marine Laboratory, Department of Marine Sciences, University of Gothenburg, 45296 Stromstad, Sweden; ^4^ Laboratory of Genetics, University of Wisconsin-Madison, Madison, WI, USA; ^5^ cE3c – Centre for Ecology, Evolution and Environmental Changes, Faculdade de Ciências, Universidade de Lisboa, Campo Grande, 1749-016, Lisboa, Portugal; ^6^ Ecology and Evolutionary Biology, School of Biosciences, University of Sheffield, Western Bank, Sheffield S10 2TN, UK; ^7^ Department of Biology, University of Fribourg, Chemin du Musée 10, CH-1700 Fribourg, Switzerland; ^8^ Department of Ecology, Environment and Plant Sciences, Science for Life Laboratory, Stockholm University, 106 91 Stockholm, Sweden

**Keywords:** arrangement, associative overdominance, balancing selection, introgression, inversion

## Abstract

Supergenes offer spectacular examples of long-term balancing selection in nature, but their origin and maintenance remain a mystery. Reduced recombination between arrangements, a critical aspect of many supergenes, protects adaptive multi-trait phenotypes but can lead to mutation accumulation. Mutation accumulation can stabilize the system through the emergence of associative overdominance (AOD), destabilize the system, or lead to new evolutionary outcomes. One outcome is the formation of maladaptive balanced lethal systems, where only heterozygotes remain viable and reproduce. We investigated the conditions under which these different outcomes occur, assuming a scenario of introgression after divergence. We found that AOD aided the invasion of a new supergene arrangement and the establishment of a polymorphism. However, this polymorphism was easily destabilized by further mutation accumulation, which was often asymmetric, disrupting the quasi-equilibrium state. Mechanisms that accelerated degeneration tended to amplify asymmetric mutation accumulation between the supergene arrangements and vice-versa. As the evolution of balanced lethal systems requires symmetric degeneration of both arrangements, this leaves only restricted conditions for their evolution, namely small population sizes and low rates of gene conversion. The dichotomy between the persistence of polymorphism and degeneration of supergene arrangements likely underlies the rarity of balanced lethal systems in nature.

This article is part of the theme issue ‘Genomic architecture of supergenes: causes and evolutionary consequences’.

## 1. Introduction

Understanding the forces that maintain genetic variation despite the eroding forces of drift, directional selection and recombination is one of the central problems of evolutionary biology. Supergenes, tightly linked sets of loci that underlie distinct complex phenotypes [1,2], represent some of the most spectacular examples of long-term balanced polymorphisms in nature. Although the genetic architecture of supergenes does not always include chromosomal rearrangements, these are frequently present [3]. We, therefore, refer to different variants of a supergene as arrangements from here on. The reduced recombination between supergene arrangements allows for unique combinations of alleles to be maintained in linkage disequilibrium, thus allowing for the maintenance of complex polymorphism in the population. However, the origin and maintenance of multiple supergene arrangements and their associated phenotypes within populations are still poorly understood [3–5].

Reduced recombination not only allows for the maintenance of multi-locus complexes that define supergenes but also puts them at risk of degeneration. The reduction in recombination generates a pseudo-population substructure; the effective population size for each arrangement in the supergene region is reduced as compared to the rest of the genome. The local decreases of effective recombination rate between supergene arrangements and of effective population size diminish the efficacy of purifying selection and can lead to the accumulation of deleterious alleles [3,6,7]. Indeed, recent theoretical and empirical work has shown that supergene systems are prone to such mutation accumulation [3,7–9]. This degeneration could destabilize the system, causing one of the arrangements (and therefore the polymorphism) to be lost. Alternatively, mutation accumulation might cause either one or both arrangements to be lethal in the homozygous state, but the polymorphism persists due to the higher fitness of supergene heterokaryotypes (i.e. individuals possessing two distinct arrangements).

These alternative outcomes correspond to ‘half lethal’ and ‘balanced lethal’ systems. In a half-lethal system, the load imposed by lethality of one supergene homokaryotype is low, because the frequency of this arrangement is expected to be low. In a balanced lethal system, only adults possessing two distinct arrangements (i.e. heterokaryotypes) are viable [10,11]. According to the rules of Mendelian inheritance, 50% of the next generation will be homozygous for either one or the other arrangement and thus inviable, meaning that half of the offspring are lost every generation. While it seems counterintuitive that such a huge genetic load could ever evolve under natural conditions, it has done so repeatedly across the tree of life, with examples in plants (e.g. *Isotoma* [12], *Rhoeo* [13] and *Gayophytum* [14]), insects (*Drosophila tropicalis* [15]) and vertebrates (*Triturus* newts [11,16]). The degeneration of supergenes by mutation accumulation provides a potential path by which a supergene system might evolve into a balanced lethal system, a mechanism that has received very little theoretical attention (but see [17,18]).

Recently, Berdan and colleagues have shown that evolution of an inversion polymorphism can lead to the formation of a balanced lethal system [8]. Since their focus was on the evolution of polymorphic inversions, the authors assumed that the inversion itself was inherently overdominant (*sensu stricto*), facilitating its invasion. Here, we relax this assumption, allowing only selection on linked variants. Thus, in our model, overdominance for the inversion arises by associative overdominance (AOD), where the heterozygote advantage experienced by a neutral variant (in this case the inversion) is due to selection on linked sites [19–22]. While AOD can be caused by overdominant alleles, it can also be generated by the masking of deleterious recessive variants in multi-locus heterozygotes. Empirical and theoretical work suggests that AOD driven by deleterious recessive variants may be a strong and common force stabilizing supergene systems [6–9,23,34].

AOD may also facilitate the formation of a supergene. Indeed, empirical data indicate that introgression of an alternative arrangement might be a common mechanism through which supergene polymorphisms originate [25,26]. As compared to a single-population origin, the introgression scenario could increase the chance of generating a balanced polymorphism, as there will be AOD due to private deleterious recessive mutations (i.e. those fixed in one population but absent from the other), resulting from divergence between populations. In such a scenario, the introgressed region may also carry genetic incompatibilities, generating underdominance, that may partly outweigh AOD. Yet, in a recent study, MacPherson *et al*. showed that the presence of recessive Bateson-Dobzhansky-Muller incompatibilities does not reduce heterosis in a scenario where deleterious alleles have surfed to high frequency independently during range expansion and then were brought together by admixture [27].

Below, we explore the role of AOD in maintaining a supergene polymorphism and ask when it can pave the way toward a balanced lethal system. Specifically, we examine a case where the introgression of an inverted arrangement generates a supergene polymorphism in the focal population. We use simulations to understand the range of conditions under which a supergene can persist and potentially evolve into a balanced lethal system.

## 2. Model

We explored how admixture between populations, fixed for alternative arrangements of an inversion, could generate a supergene polymorphism via AOD. Following admixture, AOD is generated by the masking of recessive private deleterious mutations within the supergene region. We hypothesized that the extent of AOD and its evolutionary consequences should be based on four major factors:

(1) the combination of the drift load (due to private fixed deleterious mutations that become polymorphic again following introgression, [28]) and mutational load (due to polymorphic deleterious alleles) of each population prior to admixture (we will refer to their combined effect, in the post-admixture population where both classes of alleles segregate, as ‘segregation load’),
(2) the dominance coefficient of the (derived) deleterious alleles,
(3) the (reduced) recombination rate between arrangements (i.e. gene flux, [29]),
(4) the population size post-admixture (*N*).

To examine these effects, we simulated two isolated diploid populations (*P*_1_ and *P*_2_, *N_BI_* = 2500 individuals per population) using SLiM v. 2.6 [30]. The genome consisted of two chromosomes of 10 Mb (genome length *L* = 20 Mb). The values of the simulation parameters were set based on estimates from *Drosophila melanogaster*. Mutations occurred at a rate of μ = 4.5 × 10^−9^ per bp per generation [31]. Recombination rate was set to *r* = 4.80 × 10^−8^ per base pair per meiosis [32]; recombination occurred via a combination of crossing over, at a rate of *ρ* = 3.0 × 10^−8^ per base pair per meiosis [32,33], and gene conversion (GC) at a rate of *γ* = 1.8 × 10^−8^ per base pair per meiosis [34] (GC is the unidirectional transfer of genetic material from one homologous chromosome to the other [35]); *γ* is the rate of initiation of a GC event. GC track length was modelled as a Poisson distribution, with the parameter *λ* set to 500 bp [34].

To generate differences in segregation load (hypothesis 1), we used burn-ins of two different durations (*T_BI_* = 200 000 or *T_BI_* = 500 000 generations). Since the populations were isolated, the burn-in length corresponds to divergence time. Fitness was multiplicative, and all mutations were assumed to be deleterious. The magnitude of their fitness effects (|*s*|) was drawn from a Gamma (*Γ*) distribution (*α* = 0.5, *β* = 10), with fixed dominance coefficient *h*. Separate burn-ins were run for two dominance coefficients: *h* = 0 and *h* = 0.1 (hypothesis 2). Given that not all simulations used the same burn-in, stochasticity between burn-ins could affect possible metrics of interest when comparing across different values of *h* or burn-in length (see *Significant variation with evolutionary consequences exists between burn-ins* below). Initial simulations showed that this variation could even eclipse small differences in parameters. To take into account this source of variation, we generated 10 different burn-ins per parameter combination (two population sizes and two dominance coefficients, for a total of 40 burn-ins).

We modelled the genetic architecture of the supergene as a chromosomal inversion. The inversion occurred at a fixed position on chromosome 1 and encompassed 50% of its length (i.e. 25% of the genome). The inverted arrangement was assumed to be fixed in *P*_2_, while the *P*_1_ population carried the standard arrangement. Recombination in the inverted region in the heterokaryotype was restricted to gene conversion only. The second chromosome served as a control region since indirect effects of the inversion could extend beyond the inverted region to the rest of chromosome 1.

The polymorphic period for each replicate began with the migration of a single randomly chosen individual from *P*_2_ to *P*_1_ in generation 1. This migration step established the supergene system, with the inverted arrangement from *P*_2_ and the standard arrangement from *P*_1_ forming the two arrangements of the supergene. After this migration event, we only followed evolution of *P*_1_. Simulations ended when the immigrating inverted arrangement was fixed or lost, or after 200 000 generations if it remained polymorphic. For each of the 10 burn-ins per parameter combination, we ran 10 000 replicates for a total of 100 000 replicates per parameter set (over 16 parameter sets in total).

We examined the emergence of AOD and its evolutionary consequences for the inversion polymorphism. The effect of variation in gene flux (hypothesis 3) was tested by removing GC, modelled as having all recombination events occur as crossovers so that the total rate of recombination (*r*) remained constant outside the supergene. To explore the effect of a smaller post-admixture population size (hypothesis 4), we assumed that immediately before admixture P_1_ declined in size from *N_BI_* = 2500 to its new size (*N*) and stayed at that new size for the entire post-admixture period. While this meant that the population was not always at equilibrium initially for the post-admixture period, it ensured that the segregation load remained similar between scenarios as the majority of the segregation load comes from the drift load pre-admixture (from approx. 60–90%, electronic supplementary material, figure S1). We used the following post-admixture population sizes: *N* = 100, *N* = 250, *N* = 500 and *N* = 2500. Sweeps of beneficial mutations could influence supergene evolution, for example by increasing the fixation rate of deleterious alleles. Therefore, the post-admixture period differed from the burn-in period in that beneficial mutations could also occur at a 1000× lower rate than their deleterious counterparts (4.5 × 10^−12^ per bp per generation). All beneficial mutations were co-dominant (*h* = 0.5), and their fitness effects (|*s*|) were drawn from an exponential distribution with parameter *κ* = 0.001. All parameter values are summarized in table 1.

**Table 1.** Parameter values used in simulations.

parameter	value
mutation rate (*μ*)	4.5 × 10^−9^ per bp per generation
genome length (*L*)	20 Mb
recombination rate (*r*)	4.80 × 10^−8^ per bp per meiosis
gene conversion initilization rate (*γ*)	0 or 1.8 × 10^−8^ per base pair per meiosis
gene conversion tract length (*λ*)	500 bp
burn-in length (*T_BI_*)	200 000 or 500 000 generations
dominance coefficient (*h*)	0 or 0.1
population size pre-admixture (*N_BI_*)	2500
population size post-admixture (*N*)	100, 250, 500 or 2500

We used a full block design, varying two post-burn-in parameters (GC, population size) for each of the four combinations of burn-in parameters. This design was performed for only two values of *N* (100, 2500). To further explore the relationship between population size and the emergence of balanced lethals, we considered two additional post-admixture population sizes (*N* = 250 and *N* = 500). These simulations were done for both burn-in lengths, but both GC and *h* were kept at zero, as these factors were found to be critical for the emergence of balanced lethals using our initial full block design (see *Balanced lethals can only evolve in a highly restricted parameter space* below). Finally, we examined the effects of beneficial mutations by running one set of parameters (*h* = 0, GC = 0, *N* = 2500, *T_BI_* = 200 000) in the presence and absence of beneficial mutations.

To reduce computational time, we did not simulate neutral mutations. We simulated allelic content only in 200 kb of the 20 Mb genome, divided into 5 kb segments, uniformly distributed along the genome. Recombination could occur anywhere in the genome, but deleterious and beneficial mutations only occurred in regions where we simulated allelic content. Finally, we scaled our parameters by a factor of 10, keeping *L*μ and *Lr* constant, so that evolution occurred at an accelerated rate (a common practice; e.g. see [36]).

Since we assumed a constant population size (Wright-Fisher population, with the exception of the population size reduction event), only relative fitness values were considered. More precisely, fitness in SLiM is relative to the fitness of an individual with none of the currently segregating mutations (mutations, both beneficial or deleterious, that fix are removed from the model completely) [30]. This is what we refer to as fitness throughout this paper.

## 3. Results and discussion

### (a) Significant variation with evolutionary consequences exists among burn-ins

The drift and mutational loads of the populations, following the burn-in, were affected by dominance and burn-in length. As expected, the number of fixed differences between populations increased with burn-in length, but the number of segregating mutations remained stable (electronic supplementary material, figure S2). Mutations accumulated in a linear fashion, with the 500 000 generation burn-in having approximately 2.5× more fixed mutations than the 200 000 generation burn-in. Increasing the dominance coefficient (*h*) strongly decreased the number of segregating deleterious alleles (electronic supplementary material, figure S2B). Additionally, increasing the dominance coefficient slightly decreased the average number of fixed mutations, although the distributions overlapped (electronic supplementary material, figure S2A). Thus, by simply picking randomly a single burn-in for each value of *h*, there was a non-negligible chance that the average trend observed would be reversed, highlighting the necessity of using multiple burn-ins. These effects were mirrored for segregation load (electronic supplementary material, figure S3).

**Figure 1.**
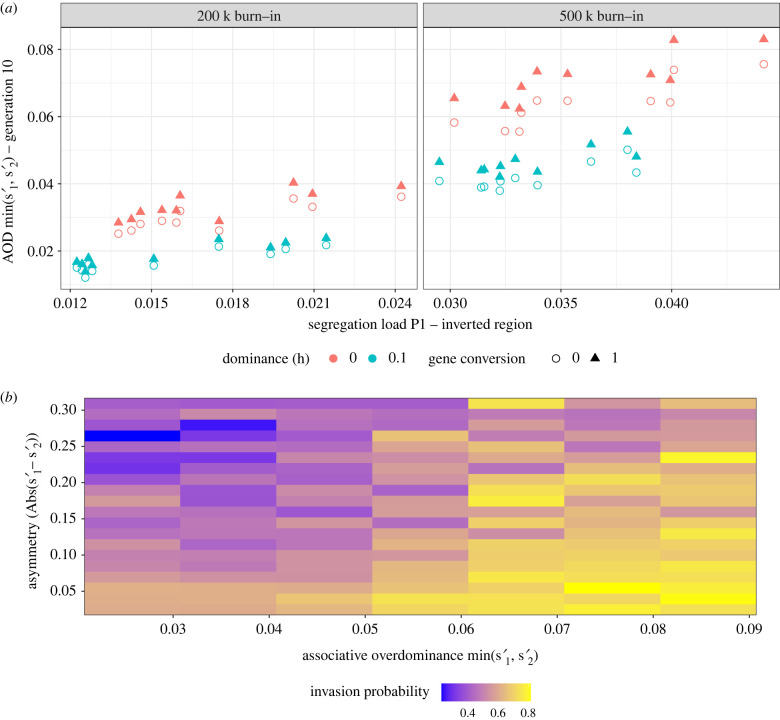
Associative overdominance (AOD) generated from segregation load can facilitate invasion of a supergene. (*a*) AOD correlates with segregation load. Shown is the average AOD at generation 10, across replicates, for each burn-in (200 000 or 500 000 generations), compared to the segregation load of the P1 population estimated from the burn-in. Colours indicate the dominance coefficient (0-red, 0.1-blue) and shape indicates the level of gene conversion (circle - absence, triangle - presence). The data shown are for *N* = 2500. (*b*) Both symmetry and strength of AOD contribute to the probability of invasion. Each tile represents a specific parameter space (±0.005 change in AOD and ±0.0075 change in symmetry (absolute $\left(s_{1}^{\prime }-s_{2}^{\prime }\right)$)). Colouring represents the probability that a simulation from that parameter space resulted in invasion. The numbers of obervations per tile differ, but all are greater than 20. The data shown are for *N* = 100, GC = 0 and *h* = 0. (Online version in colour.)

We calculated AOD following Ohta [19] as the difference in fitness between the supergene heterozygote and the fitter of the two homozygotes, $s^{\prime }=\min(s_{1}^{\prime },\;s_{2}^{\prime }),$ where

$$s_{1}^{\prime }=1-\frac{{\bar{W}}_{AA}}{{\bar{W}}_{AB}},$$

and

$$s_{2}^{\prime }=1-\frac{{\bar{W}}_{BB}}{{\bar{W}}_{AB}},$$

and where *A* denotes the inverted arrangement (originating in *P*_2_) and *B* the standard arrangement (originating in *P*_1_). Positive values of *s′* indicate that the heterokaryotype is fitter than either homokaryotype (i.e. the presence of AOD, with higher values resulting in stronger balancing selection) and negative values indicate the reverse. The segregation load of the supergene region, especially in *P*_1_ (the focal population), was strongly correlated with the strength of AOD at the beginning of the simulation (figure 1*a*), confirming hypothesis 1. Segregation load in *P*_2_ was less impactful (electronic supplementary material, figure S4), because AOD is defined by the fitter of the two homokaryotypes which was typically the standard homokaryotype from *P*_1_. Variation in segregation load and in the strength of AOD was found across parameter combinations, as well as among burn-ins within parameter combinations (figure 1*a*).

The strength of AOD at the beginning of the simulation partially predicted the probability of invasion (figure 1*b*). We considered that the inverted arrangement had successfully invaded if it was still present in *P*_1_ after *N* generations. As AOD increased, so did the probability of invasion. This is in line with theoretical predictions that AOD should aid supergene invasion [37].

### (b) Changes in AOD over time; causes and evolutionary consequences

AOD is determined by the segregation loads of the two supergene arrangements. Its strength is therefore expected to vary as the allelic contents of the supergene arrangements evolve. Nei *et al.* found that AOD could promote the invasion as well as the fixation of a new inversion in a single isolated population [38]. Yet, in many circumstances the advantage conferred by AOD might only be transient as the inverted arrangement will accumulate recessive deleterious mutations over time, which might in turn prevent the fixation of the inverted arrangement [39]. In our model, where the inversion was introduced by migration, invasion probability was also dependent on the symmetry of segregation loads of the two supergene arrangements, in addition to the strength of AOD (figure 1*b*). Two random processes drove the asymmetry of the two segregation loads: stochastic mutation accumulation in *P*_1_ and *P*_2_ and random sampling of the immigrating individual. The importance of symmetry is consistent with results from single-locus overdominance models, which show that polymorphism is more likely to be maintained under symmetrical overdominance because drift is less likely to cause the loss of a polymorphism maintained at intermediate frequency [40,41]. A critical difference between AOD and single-locus overdominance is that AOD can easily change over time [39], while the fitness overdominance at a single locus is generally assumed to be a fixed property of the two interacting alleles.

Large regions of the genome, prone to mutation accumulation due to reduced recombination, are likely to generate strong AOD [8,9]. The rate of mutation accumulation in either of two supergene arrangements is tied to both the effective recombination rate and the relative strengths of drift and purifying selection (correlated with the number of copies of the arrangement) [8,42]. While the mutation rate obviously also affects mutation accumulation [43,44], it is unlikely to be different between arrangements. A large difference between the fitnesses of supergene arrangement homozygotes results in a large frequency difference between the major (more frequent) and minor (less frequent) supergene arrangements. This means that the effective population size of the minor arrangement is (much) smaller compared to the major. This, in turn, translates into purifying selection being far less effective in the minor arrangement, which leads to an increase in mutation accumulation [8]. This has two consequences: first the relative fitness of the major arrangement will increase even more, further increasing its frequency, and therefore the efficacy of purifying selection, in a feedback loop. Second, if mutations are only partly recessive, mutation accumulation in the minor arrangement will also decrease the fitness of the heterokaryotype and thus reduce AOD. These processes drive the frequency of the minor arrangement down, increasing its chance of being lost by drift.

Together, our results point toward the initial symmetry of the mutational load of the two supergene arrangements as being a key factor determining the fate of the supergene polymorphism (figure 1*b*). A second critical factor is the magnitude of AOD, which is reflected in the fitness differential between the major arrangement and the heterokaryotype, normalized by the mean fitness of the heterokaryotype. An increase in the dominance coefficient is expected to reduce both the fitness differential as well as the mean fitness of the heterokaryotype; as predicted, we observed a negative relationship between the dominance coefficient and the probability of invasion (hypothesis 2; electronic supplementary material, figure S5).

**Figure 2.**
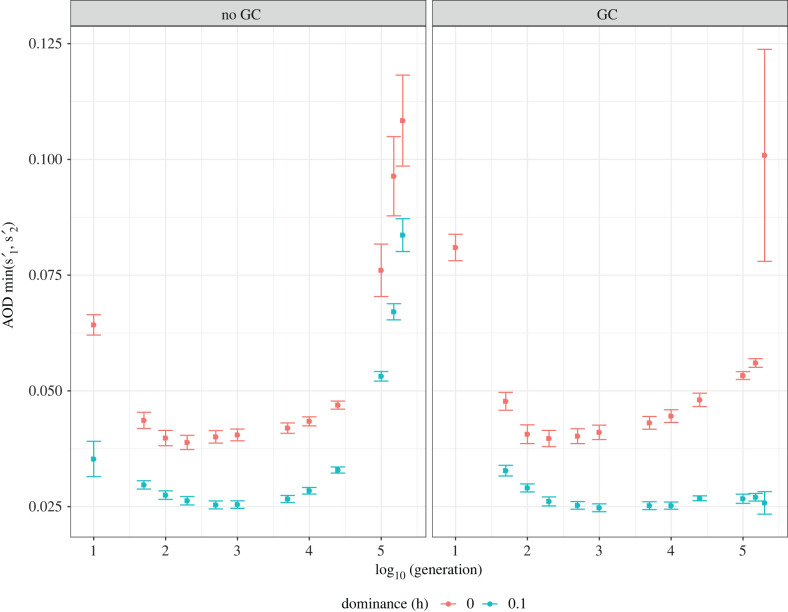
Associative Overdominance (AOD) changes in a non-monotonous manner over time. Shown is the evolution of AOD over time, only for simulations where the polymorphism lasted 200 000 generations (*N* = 2500, Burn-in = 500 000 generations). AOD for each generation is calculated as an average per burn-in, per parameter set. Error bars represent standard error between burn-ins. Colours indicate the dominance coefficient (0-red, 0.1-blue) and facets indicate the level of gene conversion (GC; presence or absence). (Online version in colour.)

The AOD present following admixture readily changed in our model: it generally decreased in the beginning of the runs and then increased again over time (figure 2). This remained true even when focusing only on simulations where the population remained polymorphic to the end, which indicates that the increase was not due to a sieving effect (i.e. cases with weak AOD were removed at early time points, leaving only cases with strong AOD at later time points). In addition, the initial decrease in AOD occurred even in the absence of GC and with fully recessive deleterious mutations, excluding the possibility that the mutational load of the introgressing arrangement decreased via gene flux and purifying selection. Thus, this initial decrease was likely due to the erosion of linkage disequilibrium between the supergene arrangement from *P*_2_ and the rest of the *P*_2_ genome. Initially, the entire chromosome 1 (50% of the genome) contributed to overdominance, but, over time, recombination would have reduced this to the supergene alone. Indeed, consistent with this expectation, the drop in AOD corresponded to both a drop in heterokaryotype fitness and a slight increase in the fitness of the inversion homokaryotype (electronic supplementary material, figure S6). A possible impact of beneficial mutations was tested by additional simulations, done in the absence of beneficial mutations, which produced the same qualitative drop in AOD in the early generations (electronic supplementary material, figure S7). After the initial decrease, AOD increased in all runs that remained polymorphic over 200 000 generations, due to mutation accumulation. This increase was stronger in the absence of GC (hypothesis 3), with fully recessive deleterious mutations (hypothesis 2), and in smaller populations (hypothesis 4; electronic supplementary material, figure S8).

### (c) Maintenance of supergene polymorphism can be accomplished in three different ways

To understand the fate of the supergene and its possible long-term maintenance, we focused on cases where the polymorphism was maintained over the full simulation period (i.e. 200 000 generations). We examined fitness across karyotypes at the final generation, considering individuals with fitness values less than 0.01 to be inviable and individuals with fitness values greater than 0.01 to be viable. We identified three qualitatively different outcomes:

(1) both homokaryotypes remained viable (${\bar{W}}_{BB}>0.01$ & ${\bar{W}}_{AA}\;>\;0.01$),
(2) one homokaryotype became inviable, while the other remained viable (i.e. a ‘half-lethal’ system; ${\bar{W}}_{BB}<0.01$ & ${\bar{W}}_{AA}>0.01$ or ${\bar{W}}_{AA}<0.01$ & ${\bar{W}}_{BB}>0.01$), and
(3) both homokaryotypes became inviable and only heterokaryotypes contributed to subsequent generations (i.e. a balanced lethal system; ${\bar{W}}_{BB}<0.01$ & ${\bar{W}}_{AA}<0.01$).

At generation 200 000 we only observed outcome 1 in large populations (*N* = 2500); it was negatively correlated with GC and positively correlated with *h* and divergence time before introgression (supporting hypotheses 1–4; electronic supplementary material, figure S9). Outcome 2 occurred almost exclusively in large populations (*N* = 2500; 99.99% of cases), without GC (99.92% of cases), with fully recessive mutations, and was positively correlated with divergence time (electronic supplementary material, figure S10A). Finally, outcome 3 (a balanced lethal system) only occurred in the small populations (*N* = 100) and was negatively correlated with *h* and positively correlated with divergence time (electronic supplementary material, figure S10B). Overall, partially recessive deleterious mutations, GC, or both, reduced the chance of the supergene remaining polymorphic for up to 200 000 generations (i.e. at least 80 N generations). The removal of beneficial mutations did not qualitatively change the probabilities of the different outcomes (electronic supplementary material, figure S11). The probabilities of all outcomes for all parameter sets at generation 200 000 are given in electronic supplementary material, table S1. Changes between outcomes at generations 100 000, 150 000 and 200 000 (i.e. persistence of outcomes) are shown in electronic supplementary material, table S2.

**Figure 3.**
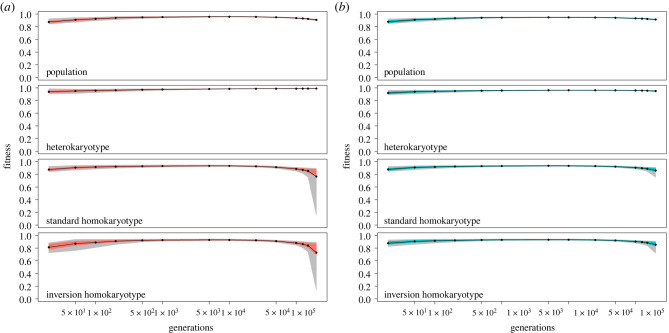
Evolution of fitness when all karyotypes remain viable for different values of *h* ((*a*) *h* = 0, (*b)* *h* = 0.1). Mean fitness of the population, relative fitness of the heterokaryotype, relative fitness of the standard homokaryotype and relative fitness of the inversion homokaryotype are shown. Time is always shown on a log scale; actual time points where data were collected were generations: 10, 50, 100, 200, 500, 1000, 5000, 10 000, 25 000, 50 000, 100 000, 125 000, 150 000 and 200 000. Only data from *N* = 2500 runs that lasted 200 000 generations are shown. (Online version in colour.)

In order to interpret these patterns, we further explored how fitnesses changed over time in cases where the polymorphism persisted. When both homokaryotypes remained viable (outcome 1), there was an initial increase in fitness of all three karyotypes (since fitness is expressed relative to a genotype without any of the segregating mutations, figure 3). In our simulations there were three ways for either homokaryotype to increase in fitness: (i) purging of deleterious mutations via gene flux, (ii) reducing linkage disequilibrium with the rest of the genome, therefore lowering the mutational load generated by private mutations, and (iii) accumulation of beneficial mutations. Recombination could occur within arrangements via crossing over and GC, or between arrangements via GC only. The introgressed inverted arrangement could not purge its initial mutational load by recombination within arrangements, since all inverted arrangements shared the same set of deleterious alleles. We observed increases in fitness for both homokaryotypes in the presence and absence of GC. Additional analysis in the absence of beneficial mutations generated qualitatively the same pattern (electronic supplementary material, figure S12). Overall, this indicates that the initial increase in fitness observed was due to the lessening of linked mutational load generated by private deleterious alleles outside the inversion (figure 3).

After the initial increase, fitness of the homokaryotypes plateaued, before decreasing again. This decrease was due to the accumulation of deleterious mutations, which did not affect the fitness of the heterokaryotype when *h* = 0 (as previously shown in [8]). It is important to note that we only ran our simulations for 200 000 generations. To better understand the nature of outcomes where both homokaryotypes remained viable (outcome 1), we focused on all simulations that were classified as ‘outcome 1’ at generation 100 000. We then followed the fate of these specific runs after 50 000 and 100 000 additional generations. We saw that 40–85% of outcome 1 cases were lost in the next 50 000 generations. After 100 000 generations, this increased to 60–100%. In general, outcome 1 evolved toward (i) the loss of the supergene, (ii) outcome 2 (a half-lethal system; found in cases where *N* = 2,500, *h* = 0 and no GC) or (iii) outcome 3 (a balanced lethal system; found in cases where *N* = 100, *h* = 0.1, present GC and *T_BI_* = 500 000; see electronic supplementary material, figure S13 and table S2A). Based on these results, we conclude that outcome 1 is a transient state, but can persist for a rather long time (at least 1000 N in some cases). This is because weak AOD (*s′)* fails to protect the polymorphism from loss by drift while strong AOD leads towards the half-lethal or balanced lethal outcomes.

In the half-lethal outcome (outcome 2), one arrangement degraded by mutation accumulation, while the other did not (electronic supplementary material, figure S14). Initially, fitnesses of all karyotypes increased, due to the accumulation of beneficial mutations and the reduction of linkage disequilibrium between the supergene and the other *P*_2_ mutations situated outside of the supergene. After reaching a plateau, the fitness of one of the homokaryotypes dropped steeply to ‘lethal’ (fitness less than 0.01), due to the accumulation of deleterious mutations, while the other remained relatively fit (electronic supplementary material, figure S14). The fitter arrangement also accumulated mutations over time, but at a much slower rate (electronic supplementary material, figure S14). This difference is due to the feedback loop between arrangement frequency and allelic content described in Berdan *et al.* [8]. A key difference between outcome 2 and outcome 1 is that in outcome 2 there is the possibility for *s′* to increase as the fitter homokaryotype slowly degrades. However, the half-lethal state is not fully stable and the polymorphism can still be lost or the system can evolve into a balanced lethal system (electronic supplementary material, table S2B). Overall, our results indicate that AOD is able to maintain a long-term polymorphism when at least one of the homokaryotypes is inviable. Indeed, many supergene systems have a single lethal homokaryotype, for example, this situation has been thoroughly documented in the ruff [45,46] and the fire ant [47,48]. In both of these systems, critical genes were disrupted by the breakpoints of an inversion, presumably at the origin of the derived arrangement. However, half-lethal systems could also arise through the accumulation of deleterious mutations as shown here. We discuss outcome 3 below (see section *Balanced lethals can only evolve in a highly restricted parameter space*).

### (d) The degradation of supergene arrangements and the maintenance of the polymorphism via AOD are at odds

Based on the parameter space we explored, the maintenance of the supergene polymorphism over long timescales by AOD alone appears unlikely. Events occurring with a frequency below 10^−4^ were ignored. Outcomes 1 and 2 only occurred in 5 and 2 parameter combinations, respectively, out of 16. This suggests that, within our model, AOD alone is only capable of maintaining a long-term balanced polymorphism in exceptional circumstances. We hypothesize that this is due to the fact that degeneration of the supergene arrangements, the process that increases AOD, typically also leads to homokaryotype asymmetry in fitness and so to extreme arrangement frequencies that make polymorphism vulnerable to loss by drift.

To test this idea, we examined how our four investigated parameters (GC, dominance coefficient, divergence time and population size) affect critical properties (table 2). For a polymorphism to be maintained, the inverted arrangement must invade, but not fix. This creates a challenge: the cause of the necessary fitness advantage of the heterokaryotype should not also generate a fitness advantage for the inverted homokaryotype. For instance, increased divergence between populations increases both invasion and fixation probability. Stronger initial AOD means that the introgressing arrangement quickly spreads in the population, thus spending less time at low frequency, where mutation accumulation is more likely. Once the introgressing arrangement invades, and if the two arrangements are approximately equivalent in load, it is unpredictable which one is fixed or lost.

**Table 2.** Shown are the relationship between the parameter and process (yellow positive, blue negative, grey no relationship). Note that dominance coefficient refers to the coefficient for deleterious mutations only.

parameter	invasion	fixation	mutation accumulation major	mutation accumulation minor	efficacy of purifying selection	homokaryotype symmetry	magnitude of AOD
gene conversion	+	=	−	−	+	+	=
dominance coefficient	−	+	−/=	−	+	+	−
divergence time	+	+	+	+	−	=	+
population size	−	−	−	−	+	−	−

If invasion succeeds, the processes that will decide the fate of the polymorphism are the rates of mutation accumulation in the major and minor arrangements. These rates of accumulation will translate into changes in the magnitude of AOD $\left(s^{\prime }=\min(s_{1}^{\prime },s_{2}^{\prime })\right)$, as well as its asymmetry $\left(\left|{s^{\prime }}_{1}-{s^{\prime }}_{2}\right|\right)$. The magnitude of AOD (*s′*) will only be affected by mutation accumulation in the fitter arrangement, which is (almost) always the major arrangement. Thus, only mutation accumulation in the major arrangement will increase *s′* and symmetry (by decreasing the absolute difference between $s_{1}^{\prime }\;\mathrm{and}\;s_{2}^{\prime }$). Mutation accumulation in the minor arrangement will not affect *s′*, but will decrease symmetry. Due to the feedback loop between the effective population size of each supergene arrangement and mutation accumulation (discussed above in *Changes in AOD over time; causes and evolutionary consequences*), mutation accumulation is faster in the minor arrangement and accelerates as its frequency declines. The presence of the feedback loop thus makes maintenance of the polymorphism by AOD alone unlikely.

Several factors impact mutation accumulation in the major and minor arrangements and the resulting fate of the polymorphism (electronic supplementary material, figure S15). First, GC (hypothesis 3), by allowing gene flux between the two arrangements, can reduce asymmetry, promoting the polymorphism. However, it should also reduce the load of deleterious alleles in both arrangements, weakening AOD. Surprisingly, we did not find a detectable reduction of the magnitude of AOD associated with GC. This might be due to the low population-wide GC rate (*Nγ*) relative to the rate of mutation accumulation. Second, incompletely recessive deleterious mutations (hypothesis 2) are partially expressed in the heterokaryotype. This results in lower AOD, making the polymorphism more likely to be lost. However, the partial expression in the heterokaryotype also allows purifying selection to act on the mutations, especially those in the minor arrangement. This slows the rate of mutation accumulation and the build-up of the asymmetry (electronic supplementary material, figure S15). Third, population size (post-admixture; hypothesis 4) has an unexpected role here. Small population size means that maintenance of the polymorphism becomes unlikely, as drift becomes stronger, with the exception of one case: the balanced lethal system, which we discuss below. On the other hand, larger population size means that selection can act upon smaller fitness differences. With stronger purifying selection, a tiny initial asymmetry in mutation load can trigger a ‘snowball effect’, leading to the loss of one of the two arrangements, even if they start off in near-perfect symmetry. Finally, the divergence time between the two populations (hypothesis 1) may be the only factor that helps to maintain the polymorphism following invasion. A stronger initial drift load in both arrangements, generated by longer divergence time, means a stronger initial AOD, plus lower asymmetry between arrangements because of the larger sample of mutations.

Up to this point we have only considered balancing selection caused by AOD driven by deleterious recessive mutations. However, other forms of balancing selection may help to stabilize the polymorphism. Negative frequency-dependent selection and spatially or temporally varying selection are well-known forms of balancing selection found in supergene systems [1,3,5,49]. For example, *Formica* ants show an elaborate social polymorphism, where colonies can be headed by either one or multiple queens (monogyne or polygyne colonies), with social morphs further differing in many other life-history traits (related to how colonies are founded, the longevity of queens, etc.) [50,51]. Monogyne colonies (homokaryotype queens) are better at colonizing new habitat patches (i.e. free of *Formica*), whereas polygyne colonies (heterokaryotype queens) fare better in habitat patches already occupied, so spatial variation in the type of habitat available causes balancing selection on the polymorphism [50,52]. Inspired by this natural example, Tafreshi *et al*. found that such a system can persist only under restricted conditions, requiring strong fitness differences and assortative mating [53].

Another mechanism, disassortative mating, is predicted to evolve in systems with strong heterozygote advantage (e.g. AOD [54]). Indeed, there is evidence of disassortative mating in many classic supergene systems that also show signs of mutation accumulation [25,26,55–57]. For example, in the white-crowned sparrow two morphs exist (a tan and white striped one) underlain by a supergene where one arrangement has degraded so much that it exists almost exclusively in the heterokaryotypic state [26]. Strong disassortative mating helps to maintain this polymorphism. Practically all matings are between the homokaryotype (tan-striped sparrows) and the heterokaryotype (white-striped sparrows), ensuring that each generation these morphs are produced at equal ratio again.

### (e) Balanced lethals can only evolve in a restricted parameter space

**Figure 4.**
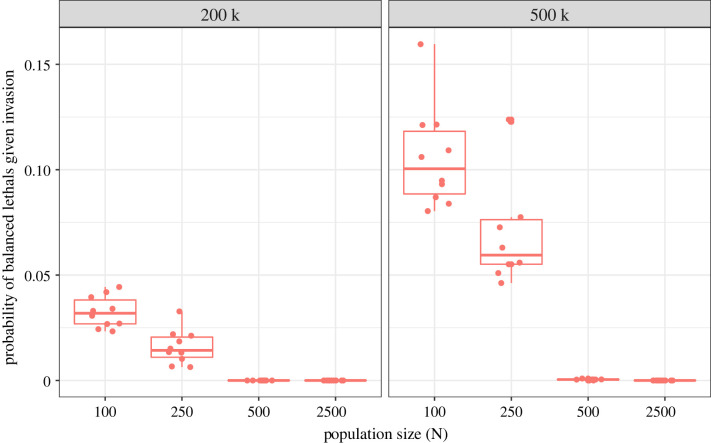
Balanced lethals evolve under restricted conditions in our simulations. Probability of balanced lethals given invasion is shown as a function of the population size in the absence of gene conversion and for fully recessive mutations (*h* = 0). Facets indicate burn-in length (200 000 or 500 000 generations). Each point is a burn-in.

The evolution of a supergene system into a balanced lethal system only happened under a stringent set of conditions in our simulations. We exclusively observed the evolution of balanced lethals in small populations. The probability of a balanced lethal outcome decreased with population size and was not observed for *N* ≥ 500 (figure 4). Our interpretation is that this is because the feedback loop between arrangement frequency and mutation accumulation is disrupted by drift in small populations [8]. The feedback loop feeds on strong differences in the efficacy of purifying selection in the major and minor arrangements (tied to differences in *N*_e_). In general, purifying selection is almost ineffective in the minor arrangement. However, in small populations, purifying selection will be less effective in general, and in the major arrangement in particular. This decreases the differential in the efficacy of purifying selection between arrangements. Additionally, arrangement frequencies will fluctuate more between generations due to drift. Together, these hamper the ‘snowballing’ of an initial asymmetry by selection, occasionally allowing for both supergene arrangements to degrade simultaneously (figure 4, electronic supplementary material, figure S16). However, in a small population, loss of the polymorphism is the most common outcome, meaning that balanced lethal systems remain rare. Critically, once a balanced lethal system had evolved, we never observed the loss of the supergene polymorphism (electronic supplementary material, table S2C). As both homokaryotypes are inviable, the polymorphism can never be lost without the extinction of the population.

Perhaps the best-known balanced lethal system is observed in the crested and marbled newts (the genus *Triturus*), with chromosome 1 existing in two forms: one with a short arm and one with a long arm. Recombination is fully supressed between forms and homokaryotypes express developmental arrest and die before hatching [58]. However, this system is not associated with particularly small population sizes in the present and this is also not seen for other known balanced lethal systems [10–12,15,16,59,60]. It is important to stress that a small population is required for the *establishment* of a balanced lethal system, but any increase in population size after that point will not be able to revert the situation. However, it would be desirable to examine past population sizes in the known examples, as a bottleneck is expected to leave a signature in the genome [61]. Now that genomic data allow inference of past population sizes, the hypothesis of a past bottleneck as a prerequisite for balanced lethal system evolution can be tested.

Another crucial factor is the dominance coefficient of the deleterious mutations. While we observed the evolution of balanced lethals when mutations were not fully recessive, the likelihood of observing this outcome was reduced relative to simulations with fully recessive mutations. This is because partially recessive deleterious mutations (i.e. 0 < *h* < 0.5) are expressed in the heterokaryotype, reducing AOD and destabilizing the polymorphism. By contrast, the presence of GC did not seem to affect the outcome in line with the fact that it had little impact on AOD overall. The generality of these effects will need to be tested using a wider range of parameter values.

For the balanced lethal systems that have been described, the genetic content of the supergene is not yet known [10–12,15,16,59,60]. In particular, we still do not know whether this is due to a few key large-effect (i.e. lethal) mutations, or the cumulative effect of mutations with smaller effect sizes (as modelled here). Large-effect mutations have been shown in half-lethal systems, indicating that this might be a more common pathway [45,46,48], but more empirical data are needed. Overall, identifying the genes present, their regulation, and the patterns of genomic variation that have evolved will be critical to understanding balanced lethal systems. Indeed, our results predict that the two supergene arrangements involved will each possess unique deleterious alleles (or even missing genes) with (almost) fully recessive effects. Fitness effects of mutations could be established via genome editing [62]. One caveat is that, post-establishment of the balanced lethal system, the two supergene arrangements will degrade further, as long as new mutations are still compensated by functional alleles in the opposite arrangement. This means we are unlikely to be able to distinguish between causal and subsequent mutations.

## 4. Conclusion

While AOD can lead to the establishment of a supergene system, under most conditions it is unlikely to maintain polymorphism over long time periods when acting alone. Supergene arrangements degenerate over time as the decrease in arrangement-specific effective population size and recombination rate leads to mutation accumulation. Initial asymmetries often mean that one of the two supergene arrangements degenerates more rapidly, which makes the supergene polymorphism sensitive to loss through drift. As AOD alone is unlikely to maintain the supergene over long time scales, other forms of balancing selection, or disassortative mating, might be key to maintaining supergene polymorphisms. This matches empirical evidence from multiple supergene systems, where combinations of different selective pressures are involved in the maintenance of polymorphism [3,4,55,63,64].

In our simulations, balanced lethals only evolved under restrictive conditions. This finding is in good qualitative agreement with the fact that, despite a broad representation across the tree of life, balanced lethal systems appear to be quite rare [10]. All documented balanced lethal systems are supergenes [12,15,16,59,60] but none of them has yet been studied at the genomic level. With the advent of next generation sequencing, properties associated with the evolution of balanced lethal systems can be investigated empirically. Our study therefore offers several new, testable hypotheses for balanced lethal systems:

(1) One of the two supergene arrangements has introgressed from a different population.
(2) The population bears the signature of an historical bottleneck.
(3) Each supergene arrangement possesses unique recessive deleterious mutations.

Although balanced lethal systems pose an evolutionary paradox, the theory and tools to unravel how they originate are now available.

## Data Availability

The simulation code and analysis code is available at gitlab.com/slottelab/BL_sims.
